# Supplementary material for: Biosynthesis and Characterization of Calcium Oxide Nanoparticles from Citrullus colocynthis Fruit Extracts; Their Biocompatibility and Bioactivities
Source: Materials (Basel). 2023 Mar 30;16(7):2768. doi: 10.3390/ma16072768 (PMC10096045; doi:10.3390/ma16072768)
Supplement: Supplementary file 1 [file materials-16-02768-s001.zip › materials-2260704-supplementary.pdf]

Supplementary Table S1 In vitro release of CaONPs (Qualifying Fig. 6)

Time	0.5 mg/mL	1 mg/mL	2 mg/mL	4 mg/mL
1 hour	2.31±0.01[a]	3.45±0.06[a]	3.96±0.08[a]	4.20±0.08[a]
2 hour	5.78±0.04[b]	6.97±0.05[b]	7.33±0.07[b]	7.63±1.74[b]
3 hour	7.62±0.04[d]	8.49±0.04[d]	9.44±0.12[c]	11.69±0.14[c]
4 hour	9.32±0.31[e]	10.41±0.05[g]	11.50±0.10[f]	13.20±0.25[d]
5 hour	10.28±0.07[h]	11.19±0.09[i]	12.98±0.13[i]	15.53±0.32[e]
6 hour	10.03±0.13[g]	11.19±0.06[i]	12.26±0.06[h]	15.53±0.13[e]
7 hour	9.69±0.11[f]	10.94±0.11[h]	11.85±0.18[g]	14.88±0.10[e]
8 hour	9.32±0.05[e]	9.99±0.07[f]	10.66±0.42[e]	13.48±0.28[d]
9 hour	7.61±0.02[d]	8.85±0.05[e]	10.14±0.23[d]	13.00±0.06[d]
10 hour	6.53±0.02[c]	7.28±0.03[c]	9.72±0.08[c]	11.42±0.09[c]

Value are expressed as mean ± Standard error of means (n = 3). Different superscripts in a column indicate significant (level of confidence 95%) difference among the variables calculated by DMRT.

Raw data for in vitro release of CaONPs (Qualifying Supplementary Table S1)

Time	Readings	0.5 mg/mL	1 mg/mL	2 mg/mL	4 mg/mL
1 hour	1	2.32	3.44	3.89	4.11
	2	2.31	3.39	4.04	4.27
	3	2.32	3.51	3.95	4.21
2 hour	1	5.81	6.92	7.30	8.57
	2	5.79	6.99	7.41	5.62
	3	5.74	7.01	7.29	8.70
3 hour	1	7.62	8.45	9.33	11.69
	2	7.65	8.53	9.57	11.55
	3	7.58	8.48	9.41	11.83
4 hour	1	9.29	10.41	11.56	13.41
	2	9.33	10.45	11.55	12.92
	3	9.35	10.36	11.38	13.27
5 hour	1	10.35	11.22	12.87	15.46
	2	10.29	11.27	13.12	15.88
	3	10.21	11.09	12.96	15.25
6 hour	1	10.01	11.21	12.27	15.68
	2	9.92	11.13	12.19	15.47
	3	10.17	11.24	12.32	15.44
7 hour	1	9.68	10.98	11.91	14.96
	2	9.81	11.03	11.99	14.77
	3	9.58	10.81	11.64	14.90
8 hour	1	9.31	9.94	10.78	13.62
	2	9.37	10.07	10.19	13.66
	3	9.28	9.97	11.01	13.15
9 hour	1	7.63	8.81	10.03	12.98
	2	7.59	8.90	9.99	13.07
	3	7.61	8.83	10.41	12.95
10 hour	1	6.52	7.28	9.68	11.41
	2	6.55	7.25	9.67	11.52
	3	6.52	7.30	9.81	11.33

Supplementary Table S2 Cytotoxic activity of CaONPs by MTT and WST-8 cytotoxicity assay (Qualifying Fig. 7)

Treatments	Percentage cell viability	
	By MTT assay	By WST-8 assay
CaONPs	60.67 ± 0.75^{a}	67.34 ± 1.64^{a}
CCFE	76.84 ± 1.05^{b}	77.06 ± 0.26^{b}
SynS	85.32 ± 0.75^{c}	85.60 ± 0.98^{c}
Control	99.40 ± 0.19^{d}	99.34 ± 0.38^{d}

Value are expressed as mean ± Standard error of means (n = 3). Different superscripts in a column indicate significant (level of confidence 95%) difference among the variables calculated by DMRT. CaONPs – calcium oxide nanoparticles; CCFE – *C. colocynthis* fruit extracts; SynS – synergistic solution of calcium oxide nanoparticles and *C. colocynthis* fruit extracts.

Raw data for cytotoxic activity of CaONPs by MTT and WST-8 cytotoxicity assay (Qualifying Table S3)

Treatments	Readings	Percentage cell viability	
		By MTT assay	By WST-8 assay
SynS	1	84.62	86.42
	2	86.12	84.51
	3	85.23	85.87
CCFE	1	78.01	77.33
	2	76.54	76.82
	3	75.98	77.03
CaONPs	1	61.26	67.93
	2	59.82	68.61
	3	60.93	65.49
Negative control	1	99.26	99.68
	2	99.63	98.92
	3	99.32	99.41

CaONPs – calcium oxide nanoparticles; CCFE – *C. colocynthis* fruit extracts; SynS – synergistic solution of calcium oxide nanoparticles and *C. colocynthis* fruit extracts.

Supplementary Table S3 Hemolytic activity of CaONPs (Qualifying Fig. 8)

Treatments	Hemolysis (%)
CaONPs	28.45±2.28^{c}
CCFE	10.19±0.99^{a}
SynS	16.00±1.61^{b}
Triton X-100	97.79±1.52^{d}

Value are expressed as mean ± Standard error of means (n = 3). Different superscripts in a column indicate significant (level of confidence 95%) difference among the variables calculated by DMRT. CaONPs – calcium oxide nanoparticles; CCFE – *C. colocynthis* fruit extracts; SynS – synergistic solution of calcium oxide nanoparticles and *C. colocynthis* fruit extracts.

Raw data for hemolytic activity of CaONPs (Qualifying Supplementary Table S3)

Treatments	Readings	Absorbance at 576 nm	$(A_t - A_c)$	$(100 - A_c)$	Hemolysis (%) = $(A_t - A_c) / (100 - A_c) \times 100$
CCFE	1	9.28	8.99	99.71	9.05
	2	11.16	10.79	99.63	10.87
	3	10.94	10.56	99.62	10.64
SynS	1	14.55	14.26	99.71	14.33
	2	16.41	16.04	99.63	16.14
	3	17.82	17.44	99.62	17.54
CaONPs	1	26.49	26.2	99.71	26.31
	2	31.06	30.69	99.63	30.84
	3	28.44	28.06	99.62	28.2
Triton X-100 (Standard)	1	96.11	95.82	99.71	96.1
	2	99.04	98.67	99.63	99.04
	3	98.23	97.85	99.62	98.22
PBS (Control)	1	0.29	0	99.71	0
	2	0.37	0	99.63	0
	3	0.38	0	99.62	0

CaONPs – calcium oxide nanoparticles; CCFE – *C. colocynthis* fruit extracts; SynS – synergistic solution of calcium oxide nanoparticles and *C. colocynthis* fruit extracts.

Raw data of Antimicrobial activity of CaONPs, *C. colocynthis* fruit extracts, and their synergistic solution, by DRSA assay (Qualifying Table 2)

Treatments	Readings	*M. luteus*	*V. cholera*	*V. parahaemolyticus*
CaONPs	1	7.1	12.1	10.8
	2	7.3	11.8	9.9
	3	7.6	11.5	10.3
CCFE	1	7.7	12.7	11.9
	2	8.2	12.1	12.3
	3	8.6	11.7	12.6
SynS	1	27.4	28.3	27.8
	2	26.6	28.7	27.2
	3	25.7	28.4	27.5
Streptomycin	1	24.5	29.5	28.1
	2	25.1	30.2	27.4
	3	24.7	30.9	27.3

CaONPs – calcium oxide nanoparticles; CCFE – *C. colocynthis* fruit extracts; SynS – synergistic solution of calcium oxide nanoparticles and *C. colocynthis* fruit extracts.

Raw data of Antioxidant activity of CaONPs, *C. colocynthis* fruit extracts, and their synergistic solution, by DRSA assay (Qualifying Table 3)

Treatments	Readings	Concentration			
		250 µl/ml	500 µl/ml	1000 µl/ml	2000 µl/ml
CaONPs	1	32.56	35.29	41.03	49.74
	2	30.92	36.57	40.91	50.05
	3	32.43	34.78	41.62	49.86
CCFE	1	49.83	57.66	61.84	68.63
	2	51.12	56.98	60.02	68.22
	3	48.96	58.17	62.25	67.89
SynS	1	66.87	73.72	81.26	82.43
	2	74.93	72.77	85.23	85.71
	3	65.90	74.12	79.03	83.48
BHT	1	71.48	76.19	84.78	89.07
	2	71.39	79.78	88.81	85.41
	3	71.57	74.44	80.63	93.77

CaONPs – calcium oxide nanoparticles; CCFE – *C. colocynthis* fruit extracts; SynS – synergistic solution of calcium oxide nanoparticles and *C. colocynthis* fruit extracts.
